# Long-term changes in renal function after treatment initiation and the importance of early diagnosis in maintaining renal function among IgG4-related tubulointerstitial nephritis patients in Japan

**DOI:** 10.1186/s13075-020-02320-x

**Published:** 2020-11-05

**Authors:** Haruna Arai, Soshiro Ogata, Takaya Ozeki, Kazuo Takahashi, Naotake Tsuboi, Shoichi Maruyama, Daijo Inaguma, Midori Hasegawa, Yukio Yuzawa, Hiroki Hayashi

**Affiliations:** 1 Department of Nephrology, Fujita Health University School of Medicine, 1-98 Dengakugakubo, Kutsukake-cho, Toyoake, Aichi 470-1192 Japan; 2 Preventive Medicine and Epidemiology, National Cerebral and Cardiovascular Center, 6-1 Kishibe-Shimmachi, Suita, Osaka 564-8565 Japan; 3 Department of Nephrology, Nagoya University Graduate School of Medicine, 65 Tsurumai-cho, Showa-ku, Nagoya, Aichi 466-8550 Japan

**Keywords:** Chronic kidney disease, Glucocorticoid, IgG4-related disease, IgG4-related tubulointerstitial nephritis, Renal biopsy, Renal pathology

## Abstract

**Background:**

The present study aimed to investigate associations between long-term renal function, whether IgG4-related tubulointerstitial nephritis (TIN) was diagnosed by renal biopsy at initial examination, chronic kidney disease (CKD) stage, and histological stage in patients with IgG4-related TIN.

**Methods:**

This study used a retrospective cohort design including almost all patients who underwent renal biopsy at Fujita Health University Hospital and Nagoya University or its affiliated hospitals in Aichi between April 2003 and March 2015 (*n* = 6977 renal biopsies). The primary outcome was longitudinal changes in eGFR. Main exposures were whether IgG4-related TIN was diagnosed by renal biopsy at the initial examination, CKD stage, and its histological stage. Linear mixed models were performed to examine associations.

**Results:**

Of the 6977 samples, there were 24 patients (with 201 records due to repeated measures) with IgG4-related TIN (20 men, mean age, 68.7 ± 9.7 years). They were followed up 6.6 ± 2.8 years after the renal biopsy and underwent glucocorticoid treatment. We found significant increase in eGFR from the baseline to 2 and 6 months after treatment initiation, which was maintained until 60 months. Patients initially diagnosed with IgG4-related TIN had higher eGFR from the baseline (at the start of treatment) to 60 months than those who were not. Compared with patients with CKD stage 3, patients with CKD stages 4 and 5 had lower eGFR at the baseline and other time points. Patients with histological stage B had comparatively lower eGFR at each point than stage A patients. Those mean differences of eGFR were stable from the baseline to 60 months.

**Conclusions:**

After the treatment initiation, renal function rapidly improved and maintained for a long period, even with advanced CKD stage. We showed importance of early diagnosis of IgG4-related TIN in maintaining eGFR.

## Introduction

IgG4-related disease **(**IgG4-RD) is a systemic fibroinflammatory condition characterized by the infiltration of IgG4-bearing plasma cells into affected organs and tissues [1–3]. In 2001, Hamano et al. demonstrated that the serum level of IgG4 was significantly elevated in patients with autoimmune pancreatitis (AIP) [4, 5]. In 2003, Kamisawa et al. proposed a new clinicopathological entity: IgG4-related autoimmune disease [6]. On the other hand, Mikulicz’s disease is associated with a high serum IgG4 level and infiltration of numerous IgG4-positive plasma cells in the affected glands [7]. These conditions have come to be understood as a systemic disease related to IgG4, and the name “IgG4-RD” was proposed at an international symposium in Boston in 2011 [8].

Renal disorder is a characteristic condition associated with IgG4-RD. IgG4-related kidney disease **(**IgG4-RKD) is pathologically characterized by IgG4-positive lymphoplasmacyte-rich tubulointerstitial nephritis (TIN) with fibrosclerosis [2, 9–13]. Patients with IgG4-RKD were only 0.67% (47 of 6978) of patients who underwent renal biopsies in the Japan Renal Biopsy Registry (J-RBR) between 2012 and 2013 as a nationwide registry. The reason why we pay attention to IgG4-RKD is that this occasionally progresses to chronic renal failure [14, 15]. In 2011, a working group in the Japanese Society of Nephrology (JSN) proposed diagnostic criteria for IgG4-RKD that covers renal parenchymal lesions and renal pelvic lesions [10], and Mayo Clinic proposed diagnostic criteria for IgG4-related TIN [11]. Previous studies showed characteristics of IgG4-RKD that were clinical, serological, radiographic, and histopathologic when IgG4-RKD was diagnosed [9–12].

Glucocorticoid treatment for IgG4-RKD has been shown to improve renal dysfunction as well as radiological and serological abnormalities. However, most previous studies were limited to relatively short observational periods [9, 11, 16–18] except for one, which showed a long-term clinical course of renal function in patients with IgG4-RKD [14]. Additionally, it has been uncertain whether long-term clinical courses of renal function are associated with how and whether IgG4-related TIN was diagnosed by renal biopsy at the initial examination or not (because there were patients who had renal biopsy before IgG4-related TIN became a recognized entity), chronic kidney disease (CKD) stage, and histological stage in patients with IgG4-related TIN.

The present study aimed to investigate the associations between longitudinal changes in eGFR, whether IgG4-related TIN was diagnosed by renal biopsy at the initial examination or not (initial diagnose with IgG4-related TIN), CKD stage, and histological stage with eGFR values at each time point.

## Materials and methods

### Study design and participants

This study used a retrospective cohort design using a Fujita Health University cohort and the Nagoya Kidney Disease Registry (N-KDR) cohort, which was based on almost all of the patients who underwent renal biopsy at those hospitals in Aichi prefecture between 2003 and 2015 (*n* = 6977 renal biopsies). Of the total 6977 renal biopsies, 24 patients (with 201 records due to repeated measures) were diagnosed with IgG4-related TIN by the authors of the present study. There were 6 patients who had not been diagnosed and 18 patients who had been diagnosed with IgG4-related TIN by renal biopsy at the initial examination. The details of this cohort were described in Additional file 1: Supplementary Data 1, Fig. S1.

Inclusion criteria of the present study were as follows: patients (1) who underwent renal biopsy between April 2003 and March 2015 and (2) who were diagnosed with IgG4-related TIN by the authors of the present study from medical records and renal biopsy specimen. IgG4-related TIN was diagnosed as follows. When serum IgG4 of the patients were measured, we used the diagnostic criteria for IgG4-RKD proposed by Japan [10]: They fulfilled the following: (1) elevation of serum IgG4 level (IgG4 > 135 mg/dl) and (2) the TIN features characteristic of IgG4-RKD, namely, dense lymphoplasmacytic infiltration with infiltrating IgG4-positive plasma cells > 10/high power field (HPF) and/or IgG4/IgG-positive plasma cells > 40% with fibrosis, and characteristic fibrosis surrounding nests of lymphocytes and/or plasma cells. When serum IgG4 of the patients were not measured, the diagnostic criteria for IgG4-TIN proposed by North America [11] were used in the present study. This is because serum IgG4 were rarely measured before the concept of the IgG4-related TIN was proposed. They fulfilled the following: (1) the histologic feature of plasma cell-rich TIN with increased IgG4-positive plasma cells and (2) at least one other feature from the categories of imaging, serology (elevated serum IgG4 or total IgG level), or other organ involvement. Note that there were 6 patients who had not been diagnosed and 18 patients who had been diagnosed with IgG4-related TIN by renal biopsy at the initial examination.

### Measurement of clinical data

To show characteristics of the present patients, we derived the following information when they underwent renal biopsy from their medical records: age, gender, serum creatinine (Cr), IgG, IgG4, C3, C4 levels, proteinuria, and hematuria. We also collected the following information between the renal biopsy and the last date of observation: prednisolone (PSL) dose, combined other non-steroidal immunosuppressive agents, kidney events (temporary hemodialysis (HD), maintenance HD), and relapse records from their medical records. For relapses, glucocorticoid dose was increased according to the discretion of the physician in charge to control the emerging or worsening symptoms of IgG4-RD. [19]

### eGFR

We retrospectively collected eGFR at the start of treatment (i.e., baseline) and at 1, 2, 6, 12, 24, 36, and 60 months from medical records. The eGFR was calculated based on an equation defined by the Japanese Society of Nephrology. The equation is as follows: eGFR (mL/min/1.73 m^2^) = 194 × serum creatinine^−1.094^ × Age^−0.287^ × 0.739 (if female). The equation has regularly been used in Japanese clinical settings [20].

### Definition of CKD stage and histological stage

All patients were categorized into the following CKD stages: stage 3 (eGFR 30–59 mL/min/1.73m^2^), stage 4 (eGFR 15–29 mL/min/1.73m^2^), and stage 5 (eGFR < 15 mL/min/1.73m^2^) referring to KDIGO CKD guideline 2012. Interstitial inflammation and fibrosis in IgG4-related TIN were classified according to the stage [2, 11]. Stages were defined as follows: stage A, active cellular infiltration with little fibrosis; stage B, active cellular infiltration with mild but distinct interstitial fibrosis; stage C, interstitial fibrosis dominant with mild cellular infiltration; and stage D, advanced interstitial fibrosis with little cellular infiltration. We categorized patients into the most relevant histological stage, as conflicting histological stages were present in the same specimen.

### Statistical analyses

Baseline characteristics were summarized by means (standard deviations [sd]) for continuous variables and *N* (%) for categorical variables.

To investigate the associations of diagnosis of IgG4-related TIN, CKD stage, and histological stage with eGFR values at each time point and longitudinal changes in eGFR, linear mixed models (LMM) were performed with random intercept and random slopes. In the LMMs, we modeled eGFR at the start of treatment and at 1, 2, 6, 12, 24, 36, and 60 months after as the primary outcome. Time after treatment initiation and variables representing pre- and post-treatment initiation were included in the models to investigate changes in eGFR, which were also used as the random slopes (i.e., individual changes in eGFR). Diagnosis of IgG4-related TIN (whether or not diagnosis was confirmed at the initial examination), CKD stage (3 [reference], 4, and 5), and histological stage (A [reference] and B) were modeled as exposures of interest. We included interaction terms between the time after the treatment initiation and each exposure of interest to compare changes in eGFR between groups of each exposure of interest. In the LMMs, we obtained regression coefficients and their 95% confidence intervals (CI) representing mean differences (95% CI) in eGFR and changes in eGFR between the groups of each exposure of interest. These were adjusted for age at the renal biopsy, sex, and hemodialysis in model 1. Model 2 was adjusted for the covariates in model 1, difference in the number of days between the renal biopsy and the treatment initiation, CKD stage when the interested exposure was diagnosis of IgG4-related TIN, and diagnosis of IgG4-related TIN when the interested exposure was CKD stage. Additionally, when the interested exposure was histological stage, model 2 was adjusted for covariates in model 1 and diagnosis of IgG4-related TIN. In model 2 for histological stage as the interested exposure, CKD stage and difference in the number of days between the renal biopsy and treatment initiation were not adjusted because we considered these variables as intermediate variables. Note that intermediate variables should not be adjusted because they are viewed as a form of over-adjustment when analyzing associations of independent and dependent variables [21]. We also investigated mean differences in eGFR between treatment initiation and each time point adjusted for age, sex, hemodialysis, diagnosis of IgG4-related TIN, difference in the number of days between the renal biopsy and treatment initiation, and CKD stage. The linear mixed models were performed by *lcmm* [22] package of R statistical software [23].

## Results

### Characteristics of the present patients

We summarized the baseline (at the start of treatment) characteristics of the 24 patients (with 201 records due to repeated measures) with IgG4-related TIN (20 men and 4 women; mean age, 68.7 ± 9.7 years) who followed up 6.6 ± 2.8 years (range 2.2–13.7 years) after the renal biopsy in Table 1. There were 6 patients who had not been diagnosed and 18 patients who had been diagnosed with IgG4-related TIN by renal biopsy at the initial examination. The mean values (SD) of serum creatinine level and eGFR were 3.21 (2.47) mg/dl and 26.0 (16.6) mL/min/1.73m^2^, respectively. For the CKD stage, 10 (41.7%), 7 (29.2%), and 7 (29.2%) patients were stages 3, 4, and 5, respectively. For the histological main stage, 6 (25.0%), 15 (62.5%), and 3 (12.5%) patients were stages A, B, and C, respectively. The reasons for performing renal biopsy were decreased kidney function (50.0%) and decreased kidney function with radiographic abnormalities (50.0%). Additionally, we described other characteristics of the present patients during the clinical courses (Table 2). All patients were treated with glucocorticoid (PSL initial dose: mean ± SD 36.7 ± 9.1 mg/day; 0.6 ± 0.1 mg/kg/day). At the last review, 5 patients were weaned from glucocorticoid, and 19 patients still on glucocorticoid (mean ± SD 3.7 ± 2.8 mg/day). Two patients (8.3%) required temporary hemodialysis (HD) in the early stage of the disease. There were no patients who progressed to end-stage renal disease, which demands maintenance HD. Two patients died of duodenal carcinoma and interstitial lung disease.

**Table 1:** Baseline characteristics of the present patients (*n* = 24)

	CKD stage 3	CKD stage 4	CKD stage 5
***n*** **(%)**	10 (41.7)	7 (29.2)	7 (29.2)
**Continuous variables, mean (SD)**			
**Age, years**	64.3 (11.1)	69.9 (8.4)	73.7 (6.4)
**Cr, mg/dL**	1.35 (0.22)	2.67 (0.61)	6.39 (2.22)
**eGFR, mL/min/1.73m**^**2**^	42.8 (9.8)	19.7 (3.7)	8.2 (3.5)
**IgG, mg/dL (reference range, 870–1700 mg/dL)**	4424.9 (1387.9)	4216.1 (1215.9)	3304.1 (685.6)
**IgG4, mg/dL (reference range, 5–105 mg/dL)**	1706.1 (1360.8)	659.5 (433.4)	505.2 (467.5)
**IgG4/IgG ratio**	0.38 (0.19)	0.18 (0.12)	0.14 (0.11)
**C3, mg/dL (reference range, 63–134 mg/dL)**	55.2 (37.2)	36.0 (19.8)	44.9 (33.3)
**C4, mg/dL (reference range, 13–36 mg/dL)**	12.0 (14.6)	1.8 (0.9)	6.4 (6.5)
**Categorical variables,** ***n*** **(%)**			
**Gender, male**	8 (80.0)	6 (85.7)	6 (85.7)
**Proteinuria**			
**(−)**	4 (40.0)	0 (0.0)	1 (14.3)
**(±)**	3 (30.0)	2 (28.6)	0 (0.0)
**(1+)**	3 (30.0)	5 (71.4)	5 (71.4)
**(2+)**	0 (0.0)	0 (0.0)	1 (14.3)
**g/gCr**	0.27 (0.24)	0.67 (0.63)	0.67 (0.19)
**Hematuria**			
**(−)**	7 (70.0)	4 (57.1)	3 (42.9)
**(±)**	1 (10.0)	2 (28.6)	1 (14.3)
**(1+)**	1 (10.0)	0 (0.0)	3 (42.9)
**(2+)**	1 (10.0)	1 (14.3)	0 (0.0)
**Histological main stage**			
**Stage A**	5 (50.0)	1 (14.3)	0 (0.0)
**Stage B**	3 (30.0)	6 (85.7)	6 (85.7)
**Stage C**	2 (20.0)	0 (0.0)	1 (14.3)

*Abbreviations: CKD* chronic kidney disease, *Cr* creatinine, *eGFR* estimated glomerular filtration rate

**Table 2:** Baseline characteristics and eGFR (Cr) at the time of renal biopsy, the time of treatment initiation, and each time point after treatment initiation

							eGFR, mL/min/1.73m^**2**^ (Cr, mg/dL)									
No.	Sex	Follow-up (yr)^**a**^	Primary diagnosis	Extrarenal lesions	PSL Tx (mg/day)	Duration of PSL treatment (yr)	At the time of renal biopsy	Baseline^**b**^	1 month from the baseline	2 months from the baseline	6 months from the baseline	12 months from the baseline	24 months from the baseline	36 months from the baseline	60 months from the baseline	The number of days between renal biopsy and the initiation of PSL treatment
**Cases not diagnosed by renal biopsy at the initial examination**^**d**^																
**1**	M	13.7	TIN associated with immune disorder	Thr, Ca,	25	13.8	15.4 (3.32)	12.8 (3.95)	19.8 (2.64)	21.6 (2.44)	23.6 (2.25)	26.9 (1.99)	28.0 (1.91)	26.7 (1.99)	27.8 (1.9)	32
**2**	M	7.8	TIN of Sjogren’s syndrome	Thr	30	2.5^c^	11.6 (3.24)	15.8 (3.22)	25.4 (2.08)	30.5 (1.76)	37.4 (1.46)	28.6 (1.86)	11.7 (4.19)	38.1 (1.42)	NA	− 17
**3**	F	12.2	TIN of Sjogren’s syndrome	Ly, sinusitis, extrathoracic lesion	30	7.5	37.4 (1.2)	46.3 (1.3)	55.6 (1.1)	55.6 (1.1)	55.6 (1.1)	63.5 (0.97)	78.0 (0.8)	68.2 (0.9)	64.4 (0.94)	− 96
**4**	M	9.3	TIN associated with immune disorder	Sa, Ly, Lu, Pa,	40	9.3	15.7 (3.5)	16.8 (3.29)	16.8 (3.29)	38.0 (1.56)	42.1 (1.42)	45.3 (1.32)	44.7 (1.33)	46.4 (1.28)	34.3 (1.67)	− 7
**5**	M	11.0	Low-grade lymphoma	Sa, Ly, Thr	60	0.5^c^	34.3 (1.6)	34.3 (1.6)	NA	NA	NA	51.4 (1.1)	51.2 (1.1)	51.0 (1.1)	46.0 (1.21)	− 90
**6**	M	7.8	TIN with immune complexes	Ca	40	7.8	16.6 (3.1)	16.6 (3.1)	37.3 (1.48)	37.9 (1.46)	37.1 (1.49)	34.6 (1.58)	33.3 (1.63)	35.1 (1.55)	23.3 (2.24)	− 14
**Cases diagnosed by renal biopsy at the initial examination**																
**7**	M	4.7	IgG4-related TIN	Sa, Ly, Lu, Thy, Pa	50	4.6	45.9 (1.25)	45.9 (1.25)	56.1 (1.04)	49.8 (1.16)	53.8 (1.08)	57.7 (1.01)	52.8 (1.09)	48.7 (1.17)	NA	− 23
**8**	M	7.7	IgG4-related TIN	NA	40	1.9^c^	5.4 (9.0)	5.4 (9.0)	9.2 (5.51)	12.9 (4.04)	19.1 (2.82)	19.0 (2.82)	21.7 (2.49)	21.5 (2.5)	20.5 (2.59)	− 1
**9**	M	6.9	IgG4-related TIN	NA	40	6.9	18.3 (2.86)	19.5 (2.82)	24.6 (2.18)	27.1 (2.0)	24.9 (2.16)	39.5 (1.41)	36.3 (1.52)	38.9 (1.42)	36.9 (1.48)	2
**10**	M	7.3	IgG4-related TIN	Sa, Ly, La	50	7.0	54.0 (1.11)	59.0 (1.05)	59.0 (1.05)	68.0 (0.92)	73.0 (0.86)	54.0 (1.13)	62.0 (0.99)	59.0 (1.03)	56.0 (1.07)	− 36
**11**	M	6.6	IgG4-related TIN	Ly, RPF	40	6.6	11.4 (4.35)	9.3 (5.25)	19.6 (2.65)	21.7 (2.41)	24.5 (2.16)	23.7 (2.21)	22.4 (2.33)	21.5 (2.41)	24.8 (2.1)	0
**12**	M	4.6	IgG4-related TIN	Ly	50	4.6	7.5 (6.2)	7.5 (6.2)	22.0 (2.28)	29.0 (1.79)	29.0 (1.82)	25.0 (2.04)	27.0 (1.89)	24.0 (2.08)	26.0 (1.97)	− 2
**13**	M	5.4	IgG4-related TIN	Sa, La, RPF	40	4.4^c^	12.4 (3.96)	12.4 (3.96)	28.7 (1.84)	28.4 (1.86)	29.8 (1.89)	32.2 (1.65)	36.3 (1.48)	32.4 (1.63)	29.3 (1.78)	− 12
**14**	M	4.6	IgG4-related TIN	Ly	30	4.6	26.1 (2.06)	26.1 (2.06)	35.9 (1.54)	35.9 (1.54)	34.5 (1.59)	37.6 (1.47)	39.8 (1.39)	42.7 (1.3)	NA	− 10
**15**	M	3.8	IgG4-related TIN	RPF, Ao	30	3.2	25.2 (2.08)	34.1 (1.58)	28.0 (1.89)	24.7 (2.12)	25.3 (2.07)	33.2 (1.61)	38.0 (1.42)	32.8 (1.62)	NA	− 14
**16**	F	2.8	IgG4-related TIN	Ly, Lu	25	2.8	22.7 (1.7)	22.5 (1.7)	27.5 (1.42)	33.3 (1.19)	33.0 (1.2)	34.3 (1.16)	33.5 (1.18)	33.7 (1.17)	NA	− 13
**17**	M	2.7	IgG4-related TIN	NA	30	2.7	9.3 (5.18)	6.7 (6.94)	12.1 (4.06)	16.6 (3.03)	15.7 (3.19)	19.0 (2.68)	16.6 (3.02)	NA	NA	− 5
**18**	F	2.2	IgG4-related TIN	NA	30	0.8	4.3 (7.81)	3.5 (9.44)	12.1 (2.85)	13.0 (2.83)	11.6 (3.13)	12.3 (2.98)	11.6 (3.13)	NA	NA	18
**19**	M	3.8	IgG4-related TIN	RPF, Ao	30	3.8	21.8 (2.39)	20.7 (2.51)	32.5 (1.66)	39.5 (1.39)	34.3 (1.58)	34.9 (1.55)	36.1 (1.5)	38.4 (1.41)	NA	5
**20**	F	8.1	IgG4-related TIN	Sa, La	30	8.0	27.4 (1.53)	30.4 (1.39)	41.0 (1.06)	43.2 (1.01)	49.4 (0.89)	43.5 (1.0)	66.0 (0.68)	55.0 (0.8)	51.0 (0.85)	− 19
**21**	M	7.8	IgG4-related TIN	Sa, Lu, Pa	45	7.7	49.5 (1.3)	49.5 (1.3)	87.7 (0.77)	70.5 (0.94)	84.7 (0.79)	80.3 (0.83)	83.1 (0.8)	92.6 (0.72)	80.4 (0.81)	− 13
**22**	M	7.4	IgG4-related TIN	Sa, Ly	30	7.4	44.7 (1.27)	44.7 (1.27)	51.1 (1.12)	61.1 (0.95)	62.6 (0.93)	58.4 (0.99)	63.0 (0.92)	61.8 (0.93)	55.6 (1.02)	− 4
**23**	M	6.7	IgG4-related TIN	Sa, Ly, RPF, Ca	30	6.2	55.8 (1.01)	52.7 (1.06)	63.1 (0.9)	52.7 (1.06)	65.4 (0.87)	52.5 (1.06)	53.4 (1.04)	52.1 (1.06)	NA	− 181
**24**	M	4.9	IgG4-related TIN	Sa, RPF	35	4.4	31.4 (1.69)	31.4 (1.69)	55.2 (1.01)	63.3 (0.89)	45.1 (1.21)	48.1 (1.14)	51.9 (1.06)	47 (1.15)	NA	− 13

*Abbreviations: M* men, *F* female, *Yr* year, *TIN* tubulointerstitial nephritis, *PSL Tx* initial dose of prednisolone, *eGFR* estimated glomerular filtration rate, *Cr* creatinine, *NA* not available, *Ao* periaortitis, *Ca* pericarditis, *La* dacryoadenitis, *Lu* lung lesion, *Ly* lymphadenitis, *Pa* type 1 autoimmune pancreatitis, *RPF* retroperitoneal fibrosis, *Sa* sialadenitis, *Thr* thrombocytopenia, *Thy* thyroiditis

^a^Length of time between the renal biopsy and the last date of observation

^b^The baseline was the time of treatment initiation. If eGFR at the time of treatment initiation was not available, eGFR at the renal biopsy was used

^c^These patients were re-treated because of relapse after discontinuation of glucocorticoid

^d^This was because there were patients who had renal biopsy before IgG4-related TIN became a recognized entity. Patients in this group was not diagnosed with IgG4-related TIN during the observation period of the present study

### Changes of eGFR

**Fig. 1:**
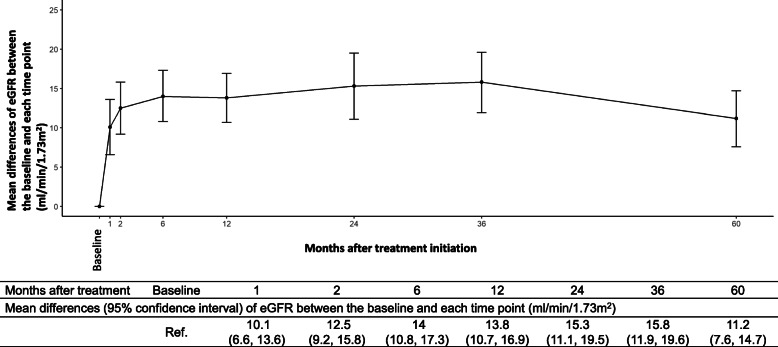
Mean differences (95% CI) of eGFR between the baseline and each time point were estimated by linear mixed models. Mean values of eGFR between the baseline and each time point were significantly different, which meant that eGFR at each time point was significantly higher compared to that at the baseline. We especially found large changes in eGFR from the baseline to 2 and 6 months after treatment initiation. The mean differences of eGFR between the baseline and from 6 to 60 were relatively stable. Abbreviations: CI, confidence interval; eGFR, estimated glomerular filtration rate

Mean differences of eGFR between the baseline and each time point were estimated by LMM and are summarized in Fig. 1. Mean values of eGFR between the baseline and each time point were significantly different. We especially found large changes in eGFR from the baseline to 2 and 6 months after treatment initiation. The mean differences of eGFR between the baseline and from 6 to 60 were relatively stable (Fig. 1).

### Changes of eGFR by diagnosis of IgG4-related TIN

We investigated mean differences in eGFR between patients who had not been diagnosed with IgG4-related TIN before it became a recognized entity (*n* = 6) and patients who had been initially diagnosed with IgG4-related TIN at the renal biopsy (*n* = 18). We used LMMs with adjustment for age, sex, and hemodialysis in model 1 and with additional adjustment for the CKD stage and difference in the number of days between the renal biopsy and the initiation of glucocorticoid treatment in model 2 (Table 3, Additional file 1: Table S1). Significantly higher values of eGFR were observed in patients who had been diagnosed with IgG4-related TIN at the renal biopsy compared to patients who had not been diagnosed (the mean difference of eGFR [95% CI] 6.1[0.7, 11.5] at the baseline, 6.1[0.8, 11.3] at 2 months, 6.0[1.3, 10.6] at 12 months, 5.7[0.9, 10.4] at 36 months). On the other hand, there were no significant difference in change rates of eGFR (i.e., eGFR slope) between the patients who had been diagnosed with IgG4-related TIN and patients who had not been diagnosed (*p* = 0.87). The mean differences of eGFR were stable during the clinical courses (baseline to 60 months), meaning cases that were able to be diagnosed maintained high eGFR throughout a long period of after treatment.

**Table 3:** Mean differences (95% CI) in eGFR at the baseline and each time point according to whether or not patients had been diagnosed at the initial examination (*n* = 24), obtained by linear mixed models

Months after treatment	Baseline^**c**^	1	2	6	12	24	36	60
**Mean differences in eGFR (ml/min/1.73 m**^**2**^**) in model 1**^**a**^								
**Cases not diagnosed by renal biopsy at the initial examination**^**d**^	Ref.	Ref.	Ref.	Ref.	Ref.	Ref.	Ref.	Ref.
**Cases diagnosed by renal biopsy at the initial examination**	9.7 (− 1.5, 20.9)	9.7 (− 1.2, 20.6)	9.7 (− 1.5, 20.9)	9.7 (− 0.9, 20.4)	9.7 (− 0.2, 19.6)	9.7 (0.5, 18.9)	9.7 (1.1, 18.3)	9.7 (1.6, 17.8)
**Mean differences in eGFR (ml/min/1.73 m**^**2**^**) in model 2**^**b**^								
**Cases not diagnosed by renal biopsy at the initial examination**^**d**^	Ref.	Ref.	Ref.	Ref.	Ref.	Ref.	Ref.	Ref.
**Cases diagnosed by renal biopsy at the initial examination**	6.1 (0.7, 11.5)	6.1 (0.7, 11.4)	6.1 (0.8, 11.3)	6.0 (1, 11.0)	6.0 (1.3, 10.6)	5.8 (1.4, 10.3)	5.7 (0.9, 10.4)	5.4 (− 1.2, 12.0)

*Abbreviations: CI* confidence interval, *eGFR* estimated glomerular filtration rate, *Ref*. reference group, *CKD* chronic kidney disease

^a^Model 1 was adjusted for age at the renal biopsy, sex, and hemodialysis

^b^Model 2 was the model 1 with additional adjustment for CKD stage and difference in the number of days between the renal biopsy and the initiation of glucocorticoid treatment

^c^The baseline was the time at treatment initiation

^d^This was because there were patients who had renal biopsy before IgG4-related TIN became a recognized entity. Patients in this group were not diagnosed with IgG4-related TIN during the observation period of the present study

### Changes of eGFR by CKD stage

**Fig. 2:**
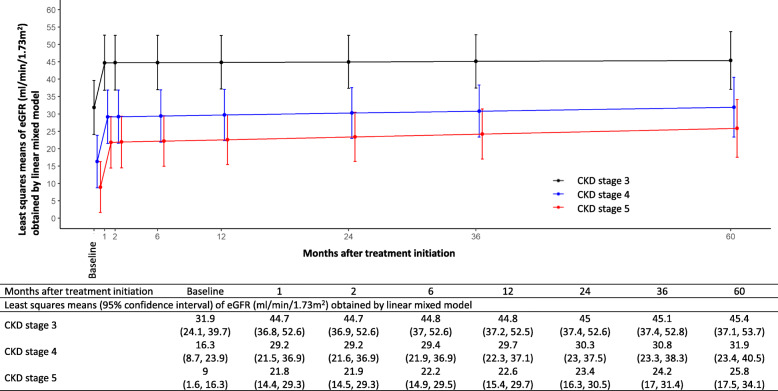
Least squares means (95% CI) of eGFR (mL/min/1.73m^2^) at baseline (i.e., at treatment initiation) and months after treatment initiation according to CKD stage 3, stage 4, and stage 5 in patients with IgG4-related TIN (*n* = 24) based on linear mixed models. Significantly lower values of eGFR were observed in patients with CKD stage 4 compared to patients with CKD stage 3. Patients with CKD stage 5 had comparatively lower eGFR than patients with CKD stage 3. Those mean differences in eGFR correspond to Table 4. On the other hand, there were no significant difference in change rates of eGFR (i.e., eGFR slope) among the patients with CKD stage 3, CKD stage 4, and CKD stage 5 (the mean difference of the monthly slope of the eGFR [95% CI], 0.0 [− 0.1 to 0.2] in CKD stage 4 and 0.1 [− 0.1 to 0.2] in CKD stage 5 compared with CKD stage 3 in model 2), meaning the mean differences of eGFR were stable during the clinical courses (baseline to 60 months). Abbreviations: CI, confidence interval; CKD, chronic kidney disease; TIN, tubulointerstitial nephritis; eGFR, estimated glomerular filtration rate

We investigated mean differences in eGFR between patients with CKD stage 3, CKD stage 4, and CKD stage 5, using LMMs with adjustment for age, sex, and hemodialysis in model 1 and with additional adjustment for whether or not they were diagnosed with IgG4-related TIN at the initial examination and difference in the number of days between the renal biopsy and the initiation of glucocorticoid treatment in model 2 (Table 4, Fig. 2). Significantly lower values of eGFR were observed in patients with CKD stage 4 compared to patients with CKD stage 3. Patients with CKD stage 5 had comparatively lower eGFR than patients with CKD stage 3. On the other hand, there were no significant difference in change rates of eGFR (i.e., eGFR slope) among the patients with CKD stage 3 (reference), CKD stage 4 (*p* = 0.62), and CKD stage 5 (*p* = 0.43).

**Table 4:** Mean differences (95% CI) in eGFR at each time point between CKD stage and histological stage obtained by linear mixed models

Months after treatment	Baseline^**d**^	1	2	6	12	24	36	60
**Mean differences (ml/min/1.73 m**^**2**^**)**								
***Model 1***^***a***^								
**CKD stage 3**	Ref.	Ref.	Ref.	Ref.	Ref.	Ref.	Ref.	Ref.
**CKD stage 4**	− 19.2 (− 25.2, − 13.1)	− 19.2 (− 25.1, − 13.2)	− 19.1 (− 25, − 13.2)	− 19.0 (− 24.6, − 13.4)	− 18.8 (− 24.1, − 13.6)	− 18.5 (− 23.5, − 13.5)	− 18.2 (− 23.5, − 12.9)	− 17.5 (− 24.7, − 10.3)
**CKD stage 5**	− 26.2 (− 33.3, − 19.1)	− 26.2 (− 33.2, − 19.1)	− 26.1 (− 33.1, − 19.1)	− 25.9 (− 32.7, − 19.1)	− 25.6 (− 32.1, − 19.1)	− 25.0 (− 31.2, − 18.7)	− 24.3 (− 30.8, − 17.8)	− 23.1 (− 31.2, − 15)
**Histological stage A**^**c**^	Ref.	Ref.	Ref.	Ref.	Ref.	Ref.	Ref.	Ref.
**Histological stage B**	− 13.9 (− 23.7, − 4.1)	− 13.9 (− 23.6, − 4.2)	− 13.9 (− 23.4, − 4.3)	− 13.8 (− 23.2, − 4.4)	− 13.6 (− 22.8, − 4.5)	− 13.4 (− 21.9, − 4.9)	− 13.1 (− 21.4, − 4.8)	− 12.6 (− 21.4, − 3.8)
***Model 2***^***b***^								
**CKD stage 3**	Ref.	Ref.	Ref.	Ref.	Ref.	Ref.	Ref.	Ref.
**CKD stage 4**	− 15.6 (− 21.6, − 9.5)	− 15.6 (− 21.5, − 9.6)	− 15.5 (− 21.4, − 9.6)	− 15.4 (− 21.1, − 9.7)	− 15.2 (− 20.6, − 9.7)	− 14.7 (− 20.1, − 9.3)	− 14.3 (− 20.2, − 8.4)	− 13.4 (− 21.3, − 5.5)
**CKD stage 5**	− 22.9 (− 29.9, − 16.0)	− 22.9 (− 29.8, − 16.0)	− 22.8 (− 29.7, − 16.0)	− 22.6 (− 29.3, − 15.9)	− 22.3 (− 28.7, − 15.8)	− 21.6 (− 28.0, − 15.2)	− 20.9 (− 27.6, − 14.1)	− 19.5 (− 28.0, − 11.0)
**Histological stage A**^**c**^	Ref.	Ref.	Ref.	Ref.	Ref.	Ref.	Ref.	Ref.
**Histological stage B**	− 13.5 (− 23.1, − 4.0)	− 13.5 (− 23, − 4.1)	− 13.5 (− 22.9, − 4.1)	− 13.4 (− 22.5, − 4.4)	− 13.3 (− 21.9, − 4.7)	− 13.1 (− 21, − 5.1)	− 12.8 (− 20.4, − 5.3)	− 12.4 (− 20.3, − 4.4)

*Abbreviations: CI* confidence interval, *eGFR* estimated glomerular filtration rate, *CKD* chronic kidney disease, *Ref.* reference group

^a^Model 1 was adjusted for age at the renal biopsy, sex, and hemodialysis

^b^Model 2 was model 1 with additional adjustment for diagnosis of IgG4-related TIN and difference in the number of days between the renal biopsy and the initiation of glucocorticoid treatment when the interested exposure was CKD stage. Model 2 was model 1 with additional adjustment for whether or not they were diagnosed with IgG4-related TIN at the initial examination when the interested exposure was histological stage

^c^Interstitial fibrosis is more clearly seen in stage B than in stage A

^d^The baseline was the time of treatment initiation

### Changes of eGFR in histological stages

**Fig. 3:**
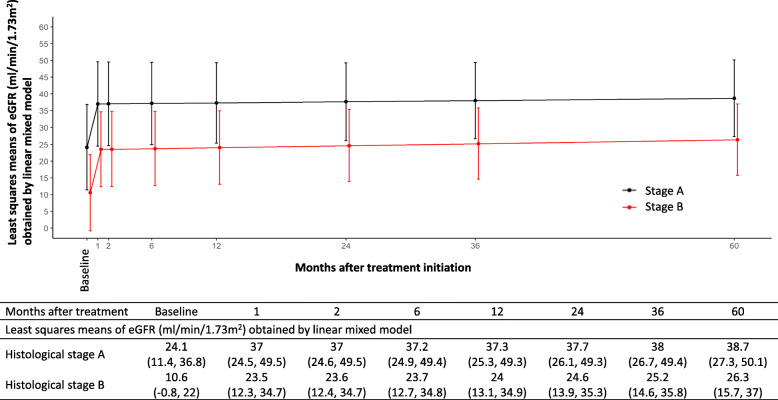
Least squares means (95% CI) of eGFR (mL/min/1.73m^2^) at baseline (i.e., at treatment initiation) and months after the treatment initiation according to histological stage A and stage B in patients with IgG4-related TIN (*n* = 24) based on linear mixed models. Significantly lower values of eGFR were observed in patients with stage B compared to patients with stage A. Those mean differences in eGFR correspond to Table 4. On the other hand, there were no significant difference in change rates of eGFR (i.e., eGFR slope) between patients with stage A and stage B (the mean difference of the monthly slope of the eGFR [95% CI] 0.0 [− 0.1 to 0.2] in model 2), meaning the mean differences of eGFR were stable during the clinical course (baseline to 60 months). Abbreviations: CI, confidence interval; eGFR, estimated glomerular filtration rate; TIN, tubulointerstitial nephritis

We investigated mean differences in eGFR between patients with stage A and stage B, using LMMs with adjustment for age, sex, and hemodialysis in model 1 and with additional adjustment for whether or not they were diagnosed with IgG4-related TIN at the initial examination in model 2 (Table 4, Fig. 3). Significantly lower values of eGFR were observed in patients with stage B compared to patients with stage A. On the other hand, there were no significant difference in change rates of eGFR between the patients with stage A (reference) and patients with stage B (*p* = 0.79).

## Discussion

Renal function rapidly improved by the two-month mark from the initial glucocorticoid therapy and was sustained for a long period by maintenance therapy. Patients who were initially diagnosed with IgG4-related TIN by renal biopsy had higher eGFR during clinical courses compared to those that were not. Compared to CKD stage 3, eGFR in CKD stage 4 and 5 was significantly lower throughout the entire period, but there was no significant difference in its change rate. Patients with histological stage B had significantly lower eGFR compared to those with histological stage A throughout the entire period, but there was no significant difference in its change rate.

Renal function rapidly improved by the 2-month mark from the initial glucocorticoid therapy and was sustained for a long period by maintenance therapy, which was supported by the following previous studies. After initiation of glucocorticoid therapy for IgG4-RKD, renal function, hypocomplementemia, and radiological abnormalities were reported to rapidly improve in 1–2 months in previous studies that had a short observational period [9, 11, 16–18]. In the retrospective study of Saeki et al. which observed the clinical course of eGFR after treatment in patients with IgG4-RKD, patients’ renal function improved and their eGFR was maintained during the time that they received maintenance glucocorticoid therapy within median 34 months [14].

In the present study, patients who were initially diagnosed with IgG4-related TIN at renal biopsy had higher eGFR during the clinical courses compared to those that were not. By appropriately diagnosing at an early stage of the disease, it can be said that early treatment can be initiated and renal function maintained compared to when it is not properly diagnosed. In the 2000s, conditions such as AIP and Mikulicz’s disease were recognized as systemic diseases related to IgG4, and the number of reports of IgG4-RD rapidly increased since around the year 2010. Moreover, before IgG4-RD became a recognized entity and spread as a disease concept, it may have been misdiagnosed as malignancy or another immune-mediated condition such as Sjögren’s syndrome.

In the present patients with CKD stages 3, 4, and 5, their eGFR were improved after the treatment. Additionally, eGFR in CKD stages 4 and 5 was significantly lower throughout the entire period than that in CKD stage 3, but there was no significant difference in its change rate. Similar results were obtained in a previous study showing that eGFR in patients whose eGFR had been less than 60 ml/min/1.73m^2^ before treatment was improved after treatment, but did not exceed 60 ml/min/1.73m^2^ [14]. The reason for this may be that patients with lower pre-treatment eGFR already have progressing fibrosis [14, 24]. In past reports, patients with eGFR<60 mL/min/1.73m^2^ before treatment had developed renal atrophy after treatment [14]. In reports evaluating histopathological findings in IgG4-related TIN from repeat renal biopsy while receiving glucocorticoid therapy, regional fibrosis developed in the interstitium, even though the area of cell infiltration decreased [16, 17]. These data might suggest that the histological fibrotic lesions correspond to the atrophic lesions demonstrated by imaging [16]. Patients with lower pre-treatment eGFR had already developed more lesions with advanced fibrosis and such lesions resulted in irreversible atrophic changes despite glucocorticoid therapy [14, 24]. Furthermore, in the present study in investigating longitudinal changes in eGFR by histological stage, histological stage B had significantly lower eGFR compared to histological stage A throughout the entire period, but there was no significant difference in its change rate. There have been no reports concerning long-term renal outcome in histological stage. This result supports past reports, which suggests improvement of renal function was limited due to the already advanced histological fibrosis and irreversible renal atrophy.

The international consensus guidance statement on the management and treatment of IgG4-RD recommends urgent treatment of IgG4-related TIN to prevent irreversible renal failure [15]. Therefore, it is important to start glucocorticoid therapy at early CKD stage and early histological stage in maintaining eGFR for a long period. Note that even in IgG4-related TIN with advanced CKD stage, eGFR has improved after glucocorticoid therapy, so it can be thought that treatment is effective.

In the present study, glucocorticoid therapy was used for all patients. Glucocorticoid therapy is common in Japan; however, other countries in North America and Europe do not perform a long-term low-dose maintenance glucocorticoid therapy [25–27], and instead, B cell depletion with rituximab and immunosuppressive agent is used with IgG4-RD patients [15, 28–30]. Therefore, further studies similar to this study are necessary in other countries.

This present study has the following strengths. First, it was based on almost all renal biopsy results of which patients with IgG4-related TIN were treated in Aichi, which could reduce selection bias. Second, we followed up on the longitudinal changes in eGFR of the IgG4-related TIN cases for a long period. Additionally, we evaluated the longitudinal changes in eGFR in CKD stage more advanced than stage 3 and in histological stage, which were not evaluated in previous studies well. This enabled us to show an even more detailed change rate of eGFR compared to previous reports.

This study has some limitations. First, the number of patients is small. This is because IgG4-RD is a rare disease, which was also showed in J-RBR as a nationwide registry with high representability in Japan. Note that the present study was based on almost all renal biopsy results in Aichi. The present study warrants further studies investigating long-term clinical course of renal function in IgG4-related TIN. Additionally, findings from the study may not be generalizable outside of Japan because treatment procedures of IgG4-RD in Japan are different from North America and Europe. Second, the treatment regimen and follow-up protocols were inconsistent among patients because of this retrospective multicenter cohort study. In this instance, 1 patient was treated after a diagnosis of non-Hodgkin’s lymphoma with cyclophosphamide, doxorubicin, vincristine, and prednisone (CHOP) chemotherapy plus rituximab. For 4 patients, an immunosuppressant (azathioprine 50 mg or mizoribine 50 mg) was added to the glucocorticoid therapy. However, as there is no consensus regarding treatment of IgG4-related TIN, further investigation is necessary. Third, relapse could not be statistically analyzed because of the small sample size, and because it is a retrospective cohort design, the definition of relapse differed per patient. However, as relapse is a significant clinical outcome, we will indicate the relapse occurrence percentage: 8 (33.3%) of 24 treated patients experienced relapse, of which 4 patients relapsed during maintenance therapy.

## Conclusions

In conclusion, renal function of patients with IgG4-related TIN was improved and maintained for a long period after glucocorticoid therapy was implemented. Compared to patients with IgG4-related TIN and CKD stage 3 or histological stage A, those with CKD stage 4 and 5 or histological stage B had lower eGFR values during the entire observational period, which suggested that early diagnosis and treatment are important in maintaining renal function. Additionally, as renal function improved after glucocorticoid therapy in all CKD stages, this can be expected even in an advanced CKD stage.

## Supplementary information

**Additional file 1: Supplementary data 1.** Description of the cohort used in the present study. **Figure S1.** Frequency and proportion of pathological diagnoses. **Supplementary data 2.** Diagnosis rate of IgG4-related TIN (2003-2008 and 2009-2015). **Table S1.** Baseline characteristics of the present patients by diagnosis or non-diagnosis of IgG4-related TIN at the initial examination.

## Data Availability

Not applicable.

## Abbreviations

AIP: Autoimmune pancreatitis
CHOP: Cyclophosphamide, doxorubicin, vincristine, and prednisone
CI: Confidence intervals
CKD: Chronic kidney disease
Cr: Creatinine
eGFR: Estimated glomerular filtration rate
HD: Temporary hemodialysis
IgG4-RD: IgG4-related disease
IgG4-RKD: IgG4-related kidney disease
LMM: Linear mixed models
PSL: Prednisolone
SD: Standard deviations
TIN: Tubulointerstitial nephritis
