# Supplementary material for: Long-term changes in renal function after treatment initiation and the importance of early diagnosis in maintaining renal function among IgG4-related tubulointerstitial nephritis patients in Japan
Source: Arthritis Res Ther. 2020 Nov 5;22:261. doi: 10.1186/s13075-020-02320-x (PMC7643351; doi:10.1186/s13075-020-02320-x)
Supplement: Supplementary file 1 — Additional file 1: Supplementary data 1. Description of the cohort used in the present study. Figure S1. Frequency and proportion of pathological diagnoses. Supplementary data 2. Diagnosis rate of IgG4-related TIN (2003-2008 and 2009-2015). Table S1. Baseline characteristics of the present patients by diagnosis or non-diagnosis of IgG4-related TIN at the initial examination. [file 13075_2020_2320_MOESM1_ESM.docx]

**Supplementary Data 1: Description of the cohort used in the present study**

This study used a retrospective cohort design based on a Fujita Health University cohort and the Nagoya Kidney Disease Registry (N-KDR) cohort that included patients who underwent renal biopsy at Fujita Health University Hospital, and Nagoya University or its affiliated hospitals as follows: Chubu Rosai Hospital, Toyohashi Municipal Hospital, Japanese Red Cross Nagoya Daiichi Hospital, Anjo Kosei Hospital, Ogaki Municipal Hospital, Kasugai Municipal Hospital, Ichinomiya Municipal Hospital, Handa City Hospital, Nagoya Kyoritsu Hospital, Tosei General Hospital, Konan Kosei Hospital, Yokkaichi Municipal Hospital, and Gifu Prefectural Tajimi Hospital.

Of the total 6977 renal biopsies at the time, there were primary glomerular disease (25.4%), IgA nephropathy (24.3%), and TIN (except IgG4-related TIN) (2.4%), and as autoimmune disease, lupus nephritis (7.6%), MPO-ANCA positive nephritis (6.1%), purpura nephritis (2.5%), anti-GBM antibody-type nephritis (0.6%), PR3-ANCA positive nephritis (0.4%). Finally, 24 patients (0.34%) were diagnosed with IgG4-related TIN throughout the entire period (between April 2003 and March 2015). For details, please see Supplemental Figure S1. Patients with IgG4-RKD were 0.67% of Japan Renal Biopsy Registry (J-RBR), a nationwide, web-based, prospective registry system including data on pathology, clinic, and laboratory of patients who underwent renal biopsies in Japan. [1,2] IgG4-related TIN is a rare disease based on the present and previous registries having high representability in Aichi and Japan.

**
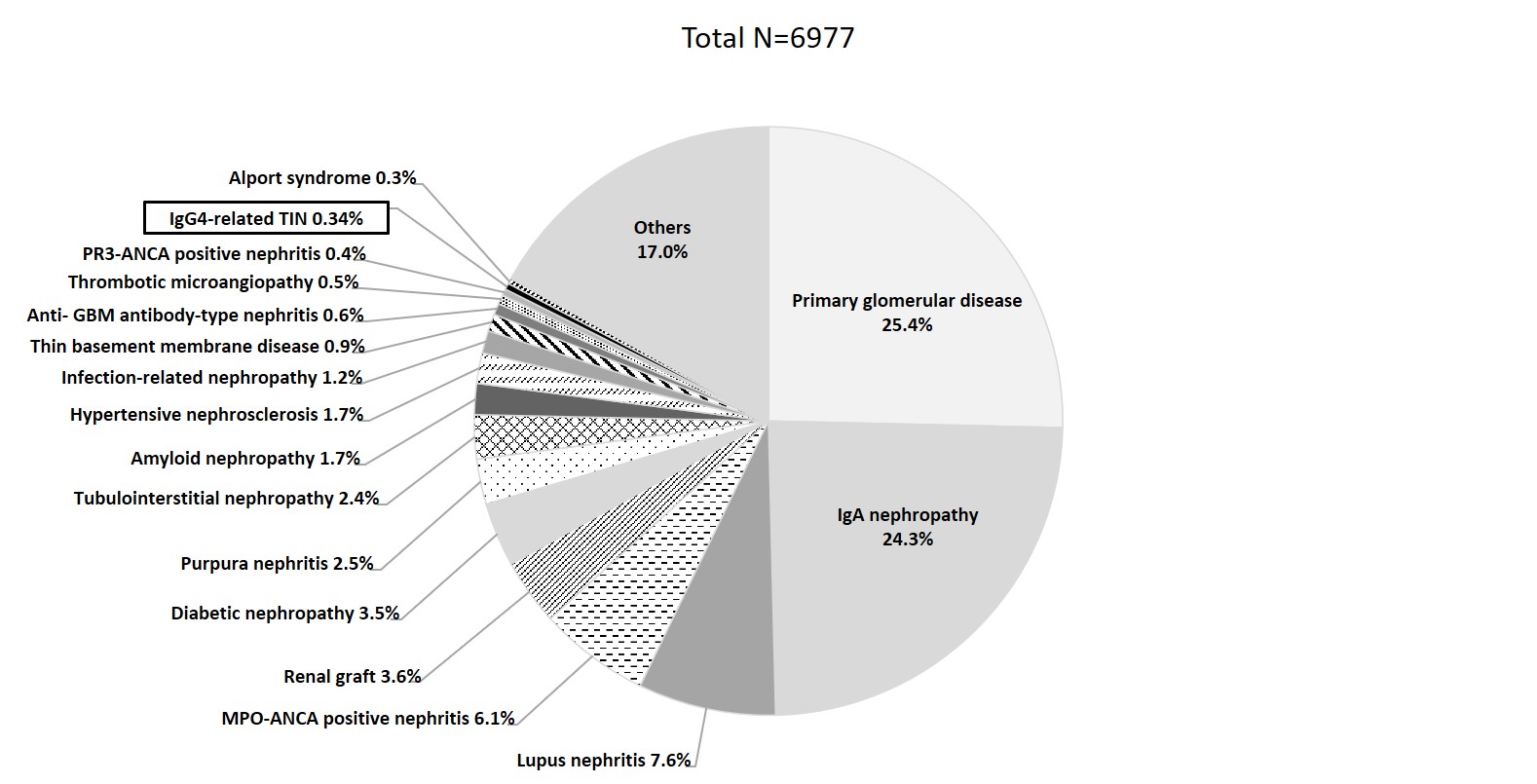
**

**Figure S1. Frequency and proportion of pathological diagnoses**

**Supplementary Data 2: Diagnosis rate of IgG4-related TIN (2003-2008 and 2009-2015)**

We investigated the rate stratified by two periods (i.e., 2003-2008 and 2009-2015) because the concept of the IgG4-RD was introduced around 2009 and its diagnosis criteria developed in 2011.

We calculated true positive rates, the percentage of actual positives correctly diagnosed with IgG4 related TIN at initial examination. Additionally, we calculated a ratio of the true positive rate between 2003-2008 and 2009-2015 using a zero-inflated negative binomial regression model by pscl package[3] of R statistical software. [4]

Of the total 2874 renal biopsies from 2003 to 2008, 7 patients were diagnosed with IgG4-related TIN during this study; however, of the 7 patients with IgG4-related TIN, only 1 patient had been diagnosed with IgG4-related TIN at the time of initial examination, meaning its true positive rate was 14.3%. The remaining 6 patients were diagnosed by TIN of Sjögren's syndrome (n=2), TIN associated with immune disorder (n=2), TIN with immune complexes (n=1), and low-grade lymphoma (n=1) at the time of initial examination before it became a recognized entity. On the other hand, of the total 4103 renal biopsies from 2009 to 2015, 17 patients were diagnosed with IgG4-related TIN during this study, and all 17 patients had been diagnosed with IgG4-related TIN at the time of initial examination, meaning its true positive rate was 100%. The true positive rate significantly increased in 2009-2015 compared to 2003-2008 (ratio of the true positive rate: 9.16, P=0.034).

Supplementary References

1. Sugiyama H, Yokoyama H, Sato H, Saito T, Kohda Y, Shinichi •, et al. Japan Renal Biopsy Registry: the first nationwide, web-based, and prospective registry system of renal biopsies in Japan. Clin Exp Nephrol. 2011;15:493–503.

2. Nakashima H, Kawano M, Saeki T, Ubara Y, Hisano S, Nagata M, et al. Estimation of the number of histological diagnosis for IgG4-related kidney disease referred to the data obtained from the Japan Renal Biopsy Registry (J-RBR) questionnaire and cases reported in the Japanese Society of Nephrology Meetings. Clin Exp Nephrol. Springer Japan; 2017;21:97–103.

3. Jackman S. {pscl}: Classes and Methods for {R} Developed in the Political Science Computational Laboratory. Sydney, New South Wales, Australia; 2017.

4. Zeileis A, Kleiber C, Jackman S. Regression Models for Count Data in {R}. J Stat Softw. 2008;27.

**Table S1. Baseline characteristics of the present patients by diagnosis or non-diagnosis of IgG4-related TIN at initial examination ^a^**

|  | **non-diagnosed cases** | **diagnosed cases** |
| --- | --- | --- |
| **n (%)** | 6 (25.0) | 18 (75.0) |
| **Continuous variables, mean (SD)** |  |  |
| **Age, y** | 65.2 (8.7) | 69.8 (9.9) |
| **Cr, mg/dL** | 2.74 (1.05) | 3.36 (2.79) |
| **eGFR, ml/min/1.73m^2^** | 23.8 (13.4) | 26.7 (17.8) |
| **IgG, mg/dL**  **(reference range, 870-1700 mg/dL)** | 4622.2 (1434.7) | 3842.1 (1124.2) |
| **IgG4, mg/dL**  **(reference range, 5-105 mg/dL)** | 214.5 (195.9) | 1122.6 (1073.9) |
| **IgG4/IgG** | 0.07 (0.08) | 0.27 (0.18) |
| **C3, mg/dL**  **(reference range, 63-134 mg/dL)** | 34.7 (22.8) | 50.6 (33.7) |
| **C4, mg/dL**  **(reference range, 13-36 mg/dL)** | 2.9 (3.0) | 8.9 (11.9) |
| **Categorical variables, n (%)** |  |  |
| **Gender, male** | 5 (83.3) | 15 (83.3) |
| **Proteinuria** |  |  |
| **(-)** | 1 (16.7) | 4 (22.2) |
| **(±)** | 0 (0.0) | 5 (27.8) |
| **(1+)** | 5 (83.3) | 8 (44.4) |
| **(2+)** | 0 (0.0) | 1 (5.6) |
| **g/gCr** | 0.88 (0.63) | 0.41 (0.29) |
| **Hematuria** |  |  |
| **(-)** | 3 (50.0) | 11 (61.1) |
| **(±)** | 2 (33.3) | 2 (11.1) |
| **(1+)** | 0 (0.0) | 4 (22.2) |
| **(2+)** | 1 (16.7) | 1 (5.6) |
| **Histological main stage** |  |  |
| **Stage A** | 2 (33.3) | 4 (22.2) |
| **Stage B** | 4 (66.7) | 11 (61.1) |
| **Stage C** | 0 (0.0) | 3 (16.7) |

a Because there were patients who had renal biopsy before IgG4-related TIN became a recognized entity.

Abbreviations: Cr, creatinine; eGFR, estimated glomerular filtration rate.
